# Association between Oral Hygiene and Metabolic Syndrome: A Systematic Review and Meta-Analysis

**DOI:** 10.3390/jcm10132873

**Published:** 2021-06-28

**Authors:** Cornelia Melinda Adi Santoso, Fera Ketti, Taufan Bramantoro, Judit Zsuga, Attila Nagy

**Affiliations:** 1 Faculty of Public Health, University of Debrecen, 4028 Debrecen, Hungary; cornelia.melinda@sph.unideb.hu (C.M.A.S.); kettifera@gmail.com (F.K.); zsuga.judit@med.unideb.hu (J.Z.); 2 Doctoral School of Health Sciences, University of Debrecen, 4028 Debrecen, Hungary; 3 Department of Dental Public Health, Universitas Airlangga, Surabaya 60286, Indonesia; taufan-b@fkg.unair.ac.id

**Keywords:** oral hygiene, dental plaque, oral bacteria, tooth brushing, interdental cleaning, dental visit, metabolic syndrome

## Abstract

Emerging evidence has linked poor oral hygiene to metabolic syndrome (MetS), but previously, no summary of evidence has been conducted on the topic. This systematic review and meta-analysis aims to evaluate the associations of oral hygiene status and care with MetS. A systematic search of the PubMed and Web of Science databases from inception to 17 March 2021, and examination of reference lists was conducted to identify eligible observational studies. A random-effects model was applied to pool the effects of oral hygiene status and care on MetS. Thirteen studies met the inclusion criteria and had sufficient methodological quality. Good oral hygiene status (OR = 0.30 (0.13–0.66); I^2^ = 91%), frequent tooth brushing (OR = 0.68 (0.58–0.80); I^2^ = 89%), and frequent interdental cleaning (OR = 0.89 (0.81–0.99); I^2^ = 27%) were associated with a lower risk of MetS. Only one study examined the association between dental visits and MetS (OR = 1.10 (0.77–1.55)). Our findings suggested that there might be inverse associations of oral hygiene status, tooth-brushing frequency, and interdental cleaning with MetS. However, substantial heterogeneity for tooth-brushing frequency and inconsistent results for oral hygiene status in subgroup analyses were observed. There was insufficient evidence for the association between dental visits and MetS. Further longitudinal studies are needed to investigate these associations.

## 1. Introduction

Metabolic syndrome (MetS), a clustering of abdominal obesity, hyperglycemia, hypertension, and dyslipidemia, represents a growing public health concern globally [1]. Although the prevalence of MetS differs depending on diagnostic criteria, age group, and ethnicity [1,2], it is estimated to affect around 25% of the world population [2,3]. MetS raises the risk of type 2 diabetes mellitus (T2DM) and cardiovascular diseases [1] and is associated with a 20% increase in healthcare costs [4].

Several risk factors for MetS have been identified. Besides socioeconomic status (SES) [5], smoking [6], diet [7], and physical activity [8], oral diseases, such as periodontal diseases and dental caries, are associated with MetS [9,10,11]. The link between oral and systemic diseases is suggested due to common risk factors, subgingival biofilm harboring Gram-negative bacteria, and periodontium serving as a cytokine reservoir [12].

Poor oral hygiene is the primary cause of common oral diseases. Accumulation of dental plaque allows bacterial growth that may lead to inflamed periodontal tissues and eventually create bacteremia and systemic inflammation [13,14]. Invading bacteria from severe caries or endodontic infections is also thought to provoke similar mechanisms [10,15,16]. Chronic low-grade inflammation underlies the development of metabolic disorders [17,18], and a study found that systemic exposure to periodontal bacteria was associated with MetS [13].

Tooth brushing and interdental cleaning, which are the main forms of oral self-care, together with regular professional care, are important measures for plaque control or removal and maintaining optimal oral health [19,20,21]. Poor oral hygiene care is associated with low-grade inflammation [22], suggesting its potential link to MetS [23]. The association of poor oral hygiene care with a higher risk of the components of MetS, such as obesity [24], diabetes [25,26], hypertension [26,27], and dyslipidemia [26,28], as well as with cardiovascular disease [14,22], has been demonstrated.

Although several epidemiological studies have reported the association of oral hygiene status [29] and care [23,30] with MetS, some studies found no such association [31,32]. To date, there has not been a systematic review conducted on the topic. A summary of evidence can provide a better understanding of the potential relationship and help healthcare practitioners deliver more targeted care. It can provide more substance for the formulation of public health programs and policies, especially strategies for the prevention and management of MetS.

The aim of our study was to systematically review the association of oral hygiene status and care with MetS and to quantify the strength of associations.

## 2. Materials and Methods

The systematic review and meta-analysis were performed according to the Preferred Reporting Items for Systematic Reviews and Meta-Analyses (PRISMA) guidelines [33]. The protocol was registered on the PROSPERO database (No. CRD42021243292) [34]. The research question was: Is better oral hygiene status or care associated with a lower risk of MetS?

### 2.1. Eligibility Criteria

The inclusion criteria were as follows: (1) The design of the study was cross-sectional, case–control, or cohort; (2) the exposure was oral hygiene status (e.g., oral hygiene index (OHI), plaque index (PI), plaque score (PSc)) or care (i.e., tooth brushing, interdental cleaning, and dental visit); (3) the outcome was MetS, clearly defined using diagnostic criteria for the condition (e.g., National Cholesterol Education Program Adult Treatment Panel III (NCEP ATP III), International Diabetes Federation (IDF), Joint Interim Statement (JIS)); (4) the study assessed the association between exposures and outcome in multiple analysis. There was no limitation on the characteristics of the study population. Animal studies, clinical trials, reviews, editorial letters, commentaries, case series, and case reports were excluded.

### 2.2. Search Strategy

A systematic search was performed on the PubMed and Web of Science databases, with the following keywords: oral hygiene, dental deposit, OHI, PI, PSc, tooth brushing, interdental cleaning, dental visit, and MetS. While no date restrictions were imposed, the language was limited to English. The last search was on 17 March 2021. Details of the search strategy can be seen in Supplementary Table S1. Examination of reference lists of eligible studies and relevant systematic reviews were also conducted to identify further relevant studies.

### 2.3. Study Selection and Data Extraction

Two authors independently screened all titles and abstracts to evaluate eligibility. Relevant studies were then examined for full-text review. Any ambiguities or disagreements were resolved by consensus. JabRef 5.2 was used during the review process.

Data from included studies were extracted independently by two authors using a data extraction form. The following information was collected: first author, publication year, study country, study design, sample size, age, gender, type of oral hygiene assessment, diagnostic criteria used for MetS, number of MetS cases, adjusted odds ratio (OR) or risk ratio (RR) with 95% confidence interval (CI), and adjustment factors. Discrepancies in data extraction were resolved by consensus.

### 2.4. Quality Assessment

Two authors independently examined the quality of included studies using the Newcastle–Ottawa Scale for cross-sectional, case–control, and cohort studies, as applicable. The three main domains examined were the selection of participants, comparability of study groups, and assessment of exposure/outcome of interest. The total scores for case–control and cohort studies were 9 points, while cross-sectional studies were 8 points [35,36]. The included studies were then categorized into high (≥7 points), moderate (4–6 points), or low (0–3 points) quality. Any disagreements were resolved by consensus.

### 2.5. Statistical Analyses

Meta-analysis was conducted separately for different types of exposure (i.e., oral hygiene status, tooth brushing, and interdental cleaning). The OR was used as the common measure for the association between oral hygiene and MetS. The reported RR was considered approximately as OR [37]. The data utilized in the meta-analysis were the estimates and the corresponding 95% CI from the most adjusted model in the studies.

The categorization of exposure varied between studies. Poor oral hygiene status or care was used as the reference group, equivalent to the highest value of OHI, PI, and PSc or the lowest frequency category of tooth brushing, interdental cleaning, and dental visits in each study. If a study classified the exposure into more than two categories, a single effect estimate was produced by combining the results of the categories using a fixed-effects (FE) model [38]. An overall pooled OR for the main analysis was calculated using a random-effects (RE) model (DerSimonian and Laird). 

Heterogeneity was assessed using the I^2^ statistic, with the value of ≥50% representing substantial heterogeneity [37,39]. Potential sources of heterogeneity were assessed using prespecified subgroup analyses by study design and country. Examination of publication bias using funnel plot and Egger’s test was only recommended if there were an adequate number of studies (>10) [40,41].

Meta-analysis was conducted using the generic inverse variance method in Review Manager (RevMan) 5.4 software (The Cochrane Collaboration, 2020) [42].

## 3. Results

### 3.1. Literature Search

**Figure 1:**
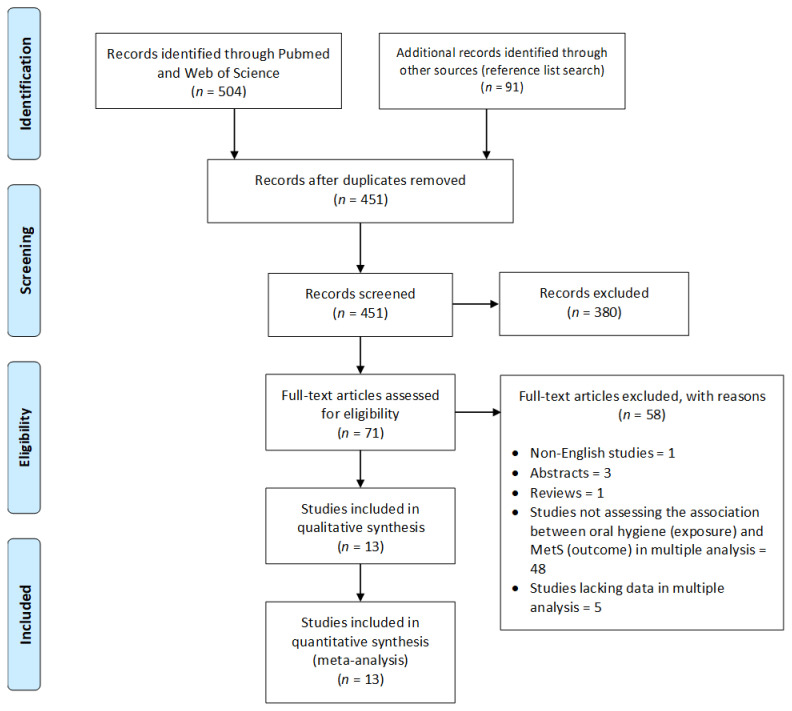
PRISMA flow diagram of the literature search and study selection [33]. MetS, metabolic syndrome.

Figure 1 shows the process and the results of study selection. A total of 595 records were identified, of which 144 were duplicates; 380 irrelevant studies were eliminated. Of the 71 studies selected for full-text review, 13 met the eligibility criteria and were included in the review and meta-analysis.

### 3.2. Characteristics of Studies

Table 1 shows the main characteristics of the included studies. They consisted of seven cross-sectional, three case–control, and three cohort studies. A study by Shearer et al. [32] examined data from a cohort study. However, because our exposure of interest (modified OHI-S) was measured simultaneously with the outcome (MetS) at age 38, we chose to consider it as cross-sectional and reported the results of their cross-sectional model. 

Eleven studies were from Asian countries, and one study each was from Finland and New Zealand. All were conducted among adult populations. Publication years ranged from 2009 to 2020, and the mean sample size was 4251.

Six studies reported oral hygiene status, six studies reported tooth-brushing frequency, two studies reported interdental cleaning, and one study reported dental visits as study factors. In the meta-analysis, a study by Tsutsumi et al. [43] was treated as two separate studies, as it reported the results independently for males and females instead of total samples. A similar approach was applied to a study by Kim et al. [44], as it provided separate data on interdental brushing and flossing.

Health examination was performed in all included studies to ascertain MetS conditions. Four studies used the NCEP ATP III criteria or its adapted version, five studies used JIS criteria, two used IDF criteria, and two used other criteria to define MetS. The most common confounders adjusted in the studies were age, gender, SES, smoking status, alcohol consumption, physical activity, and periodontal parameters. All studies reported a measure of associations as ORs, except for one study [31].

**Table 1:** Main characteristics of the 13 included studies.

Author, Publication Year	Country	Study Design	Sample Size (M, F)	Age Range	Type of Oral Hygiene	Diagnostic Criteria for MetS	Number of Cases	Statistical Analysis; Adjustments	Association
Fukui et al., 2012 [45]	Japan	Cross-sectional	6421 (M: 4944, F: 1477)	34–77	Tooth-brushing frequency (times/day)	Modified NCEP ATP III *, except the use of BMI ≥ 25 kg/m^2^ to define obesity. Treatments for raised TG and reduced HDL were not recorded.	958	Logistic regression; age, gender, smoking habit, alcohol consumption, C-reactive protein, number of teeth, periodontal parameter (PD or CAL).	OR (95% CI) Adjusted by PD: ≤1 time daily (reference) 2 times daily = 0.67 (0.57–0.78) ≥3 times daily = 0.50 (0.40–0.64) Adjusted by CAL: ≤1 time daily [reference] 2 times daily = 0.66 (0.57–0.77) ≥3 times daily = 0.50 (0.39–0.63)
Kim et al., 2013 [44]	South Korea	Cross-sectional	18742 (M: 8034, F: 10708)	≥19	Tooth-brushing frequency (times/day), use of dental floss (yes or no), use of interdental brush (yes or no)	Modified NCEP ATP III * for Asians.	5878	Logistic regression; age, gender, income, education, smoking, alcohol intake, and physical activities.	OR (95% CI) Tooth-brushing frequency: ≥3 times daily (reference) 2 times daily = 1.23 (1.12–1.34) ≤1 time daily = 1.23 (1.04–1.47) Use of dental floss: Yes [reference] No = 1.23 (1.07–1.41) Use of interdental brush: Yes [reference] No = 1.05 (0.92–1.20)
Tsutsumi and Kakuma, 2015 [43]	Japan	Cross-sectional	12548 (M: 7703, F: 4845)	30–59	Tooth-brushing frequency (times/day)	Obesity (body mass percentage ≥ 20% in men or ≥30% in women, and/or BMI ≥ 25 kg/m^2^) and at least one of the following: TG ≥ 150 mg/dL and/or low HDL < 40 mg/dL or drug for hypertriglyceridemia, SBP ≥ 130 mm Hg and/or DBP ≥ 85 mm Hg or drug for hypertension, FPG ≥ 110 mg/dL or drug for diabetes).	3624	Logistic regression; Males: age, exercise during holidays, favorite seasoning, eating soup, sugar in coffee, having an interest in losing weight, housekeeping during holidays; Females: age, favorite seasoning, worrying about job, sugar in coffee, pickles and food boiled in soy sauce, exercise during holidays, eating quickly, preparation of dinner, solving problems immediately.	OR (95% CI) Males: None (reference) 1 time daily = 0.57 (0.40–0.81) 2 times daily = 0.50 (0.35–0.71) ≥3 times daily = 0.42 (0.29–0.61) Females: ≤1 time daily (reference) 2 times daily = 0.65 (0.48–0.87) ≥3 times daily = 0.44 (0.32–0.62)
Kim et al., 2019 [46]	South Korea	Cross-sectional	8314 (M: 3860, F: 4454)	35–79	Tooth-brushing frequency (times/day)	Three or more of the following five: WC ≥ 90 cm in men or ≥85 cm in women, TG > 150 mg/dL or treatment for raised TG, HDL <40 mg/dL in men or <50 mg/dL in women or treatment for reduced HDL, SBP ≥ 130 mm Hg and DBP ≥ 85 mm Hg or antihypertensive medication, FPG ≥ 100 mg/dL or current use of antidiabetic medication.	2834	Logistic regression; age, gender, household income, education, smoking, alcohol intake, physical activity, periodontitis.	OR (95% CI) Frequency of daily tooth-brushing (continuous) = 0.887 (0.84–0.94)
Saito et al., 2019 [47]	Japan	Cross-sectional	2379 (M: 960, F: 1419)	75 and 80	Use of secondary oral hygiene products, such as dental floss or interdental brushes (none or sometimes or every day)	JIS ^ǂ^, except the use of BMI ≥ 25 kg/m^2^ to define obesity and the use of HbA1c levels ≥ 5.6% to additionally define elevated glucose. Treatments for raised TG and reduced HDL were not included.	563	Logistic regression; age, gender, smoking, exercise, weight gain, eating speed, cholesterol drug intake, community periodontal index, number of teeth.	OR (95% CI) None (reference) Sometimes = 1.19 (0.92–1.54) Everyday = 0.71 (0.55–0.92)
Shearer et al., 2018 [32]	New Zealand	Cross-sectional	836	38	Modified OHI-S (very low (0–0.5) or low (>0.5–1.0) or moderate (>1.0–1.5) or high (>1.5))	NCEP ATP III ^¤^, except the use of HbA1c ≥ 5.7% (≥39 mmol/mol) to define elevated glucose and the use of antihypertensive drugs to additionally define elevated blood pressure.	152	Logistic regression; gender, low socioeconomic status, smoking, dysglycemia, inflammatory load.	OR (95% CI) Low (reference) High = 0.95 (0.44, 2.01)
Chen et al., 2011 [48]	Taiwan	Cross-sectional	253 (M:117, F: 136)	>18	PI	Modified NCEP ATP III * for Asians, except the use of FPG ≥ 110 mg/dL or previously diagnosed T2DM to define elevated glucose.	145	Logistic regression; age, gender, education, smoking, high-sensitivity C-reactive protein, and serum albumin.	OR (95% CI) PI score (continuous) = 1.724 (1.135–2.615)
Kobayashi et al., 2012 [30]	Japan	Cohort prospective, 3-year follow-up	685 (M: 513, F: 172)	-	Tooth-brushing frequency (times/day)	JIS ^ǂ^ for Asians, except not including treatments for raised TG, reduced HDL, and elevated glucose.	99	Logistic regression; age, gender, smoking status, drinking status, breakfast eating, educational level, occupation (desk work or non-desk work), depressive symptoms, physical activity, and total caloric consumption.	OR (95% CI) ≤1 time daily (reference) 2 times daily = 0.80 (0.49–1.31) ≥3 times daily = 0.43 (0.19–0.97)
Tanaka et al., 2018 [23]	Japan	Cohort retrospective, 5-year follow-up	3722 (M: 2897, F: 825)	35–64	Tooth-brushing frequency (times/day), dental check-ups (regular or irregular)	JIS ^ǂ^ for Asians, except the use of BMI ≥ 25 kg/m^2^ to define obesity.	412	Logistic regression; age, gender, periodontal status, number of present teeth, occupational status, smoking quantity, alcohol consumption, physical activity, dietary behavior, food preference, tooth-brushing frequency, dental check-ups, and number of MetS components at baseline.	OR (95% CI) Tooth-brushing frequency: ≤1 time daily (reference) 2 times daily = 0.83 (0.65–1.05) ≥3 times daily = 0.64 (0.45–0.91) Dental check-ups: Irregular (reference) Regular = 1.10 (0.77–1.55)
Pussinen et al., 2020 [31]	Finland	Cohort prospective, 21-, 27-, 31-year follow-up	586 (M: 270, F: 316)	27–43	Presence of visible plaque (yes or no)	JIS ^ǂ^ for Europeans.	153	Poisson regression; age, gender, childhood BMI, family income, adulthood smoking (ever) and socioeconomic status (education), and interaction terms between caries and periodontal parameters.	RR (95% CI) No (reference) Yes = 1.21 (0.87–1.86)
Pham, 2018 [29]	Vietnam	Case–control (case = 206, control = 206)	412 (M: 114, F: 298)	50–78	PI (≤2.5 or 2.51–2.90 or 2.91–3.26 or ≥3.27)	JIS ^ǂ^ for Asians.	206	Logistic regression; age, gender.	OR (95% CI) ≤2.5 (reference) 2.51–2.90 = 4.81 (1.74–13.27) 2.91–3.26 = 6.12 (2.24–16.70) ≥3.27 = 7.50 (2.80–20.12)
Li et al., 2009 [49]	China	Case–control (case = 152, control = 56)	208 (M: 85, F: 123)	37–78	PI (≤1 or >1–1.5 or >1.5–2 or >2)	IDF ^§^	152	Logistic regression; age, gender, smoking.	OR (95% CI) ≤1 (reference) >1–1.5 = 4.81 (0.81–28.63) >1.5–2 = 13.06 (2.24–76.18) >2 = 47.4 (6.94–323.68)
Li et al., 2020 [50]	China	Case–control (case = 114, control = 49)	163 (M: 60, F: 103)	37–78	PI	IDF ^§^	114	Logistic regression (backward); age, gender, smoking habits, bleeding index, PD, biomarkers (serum C-reactive protein, salivary IL-6, IL-1β).	OR (95% CI) PI score (continuous) = 14.69 (5.56–38.84)

M, male; F, female; MetS, metabolic syndrome; WC, waist circumference; BMI, body mass index; TG, triglycerides; HDL, high-density lipoprotein; SBP, systolic blood pressure; DBP, diastolic blood pressure; FPG, fasting plasma glucose; HbA1c, glycated haemoglobin; T2DM, type 2 diabetes mellitus; OHI-S, simplified oral hygiene index; PI, plaque index; PD, probing depth; CAL, clinical attachment level; OR, odds ratio; RR, risk ratio; CI, confidence interval. ^¤^ The National Cholesterol Education Program Adult Treatment Panel III (NCEP ATP III) (2001) definition is any three of the following five: WC > 102 cm (>40 in) in men or >88 cm (>35 in) in women, TG ≥ 150 mg/dL, HDL < 40 mg/dL in men or <50 mg/dL in women, blood pressure ≥ 130/85 mm Hg, FPG ≥ 110 mg/dL [51]. * The modified NCEP ATP III (2005) definition is any three of the following five: WC ≥ 102 cm (≥40 in) in men or ≥88 cm (≥35 in) in women (for Asians: ≥90 cm (≥35 in) in men and ≥80 cm (≥31 in) in women), TG ≥ 150 mg/dL (1.7 mmol/L) or treatment for raised TG, HDL < 40 mg/dL (1.03 mmol/L) in men or <50 mg/dL (1.3 mmol/L) in women or treatment for reduced HDL, SBP ≥ 130 mm Hg or DBP ≥ 85 mm Hg or treatment for hypertension, FPG ≥ 100 mg/dL or treatment for elevated glucose [52]. ^§^ The International Diabetes Federation (IDF) (2005) definition is increased WC (ethnicity specific) plus any two of the following four: TG ≥ 150 mg/dL (1.7 mmol/L) or treatment for raised TG, HDL < 40 mg/dL (1.03 mmol/L) in men or <50 mg/dL (1.29 mmol/L) in women or treatment for reduced HDL, SBP ≥ 130 mm Hg or DBP ≥ 85 mm Hg or treatment for hypertension, FPG ≥ 100 mg/dL (5.6 mmol/L) or previously diagnosed T2DM [53]. ^ǂ^ The Joint Interim Statement (JIS) (2009) definition is any three of the following five: increased WC (population- and country-specific), TG ≥ 150 mg/dL (1.7 mmol/L) or treatment for raised TG, HDL < 40 mg/dL (1.0 mmol/L) in men or <50 mg/dL (1.3 mmol/L) in women or treatment for reduced HDL, SBP ≥ 130 mm Hg and/or DBP ≥ 85 mm Hg or treatment for hypertension, FPG ≥100 mg/dL or treatment for elevated glucose [54].

### 3.3. Quality Aspects of Studies

All the included studies were of moderate to high quality. One cross-sectional study, two case–control studies, and three cohort studies were of high quality. Six cross-sectional studies and one case–control study were of moderate quality. Details of the quality assessment of included studies can be seen in Supplementary Table S2.

### 3.4. Association between Oral Hygiene Status, Care, and MetS

**Figure 2:**
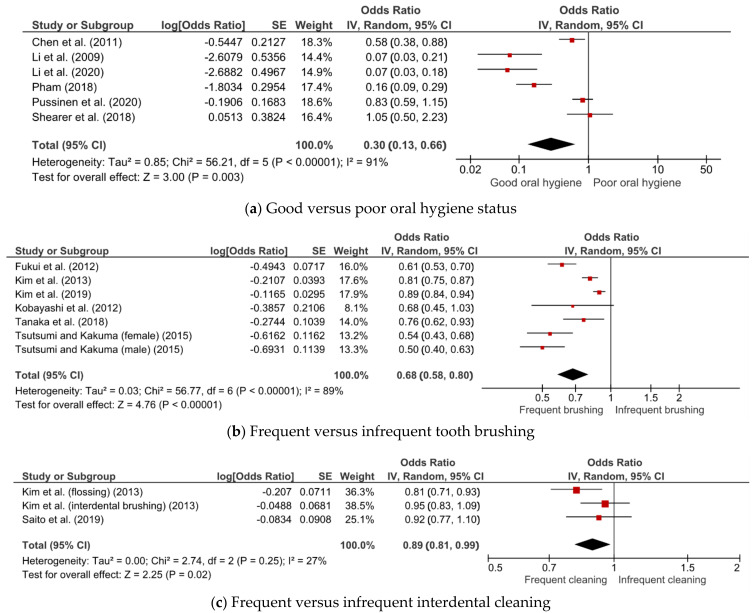
Meta-analysis of the associations of (**a**) oral hygiene status, (**b**) tooth-brushing frequency, and (**c**) interdental cleaning with metabolic syndrome.

Figure 2 shows the results of the meta-analysis of associations of oral hygiene status, tooth-brushing frequency, and interdental cleaning with MetS. Good oral hygiene (OR = 0.30; 95% CI = 0.13–0.66), frequent tooth brushing (OR = 0.68; 95% CI = 0.58–0.80), and frequent interdental cleaning (OR = 0.89; 95% CI = 0.81–0.99) were associated with a lower risk of MetS. While heterogeneity was minimal for interdental cleaning (I^2^ = 27%), there was substantial heterogeneity for oral hygiene status (I^2^ = 91%) and tooth-brushing frequency (I^2^ = 89%).

The association between dental visits and MetS was evaluated only in a study by Tanaka et al. It was found that dental visits were not significantly associated with MetS (OR = 1.10; 95% CI = 0.77–1.55) [23].

### 3.5. Subgroup Analyses

Table 2 displays the results of subgroup analysis by study design for the association between oral hygiene status and MetS. The inverse association between oral hygiene status and MetS was only observed in the subgroup of case–control studies. Subgroup analysis by study design reduced heterogeneity to less than 50%.

Table 3 shows the results of subgroup analyses for the association between tooth-brushing frequency and MetS. Frequent tooth brushing was consistently associated with a lower risk of MetS in all subgroup analyses. However, high heterogeneity was still observed among studies with a cross-sectional design. While subgroup analysis by country reduced heterogeneity, it remained above 50%.

## 4. Discussion

Our systematic review and meta-analysis investigated the association of oral hygiene status and care with MetS. Better oral hygiene status, frequent tooth brushing, and frequent interdental cleaning were associated with a lower risk of MetS. However, substantial heterogeneity for tooth-brushing frequency and inconsistent results for oral hygiene status in subgroup analyses were noted. Our review identified only one study examining the association between dental visits and MetS, and found no association [23].

While our main analysis revealed an inverse association between better oral hygiene status and MetS, the finding was inconsistent in subgroup analysis by study design. Of all studies included in the meta-analysis for oral hygiene status, only studies by Shearer et al. [32] and Pussinen et al. [31], conducted in New Zealand and Finland, respectively, did not find an association. These different findings might be due to the age of the study samples. Both studies had relatively younger samples than the other studies, which had a sample mean age of more than 50 years. The stronger influence of periodontal inflammations on cardiometabolic health may only be observed in later life [32]. Moreover, Pussinen et al. [31] reported both the adjusted RRs for MetS and β values for the number of MetS components. While the adjusted RR for the association between the presence of plaque and MetS was not significant, the β value for the association between the number of teeth with plaque and the number of MetS components was significant [31].

Our overall findings are in line with other systematic reviews and meta-analyses that demonstrated an association between oral health or hygiene and metabolic conditions [9,37]. Poor oral hygiene not only leads to dental infections, such as periodontitis, but it may also affect systemic health [55]. Periodontal bacteria in plaque, their products, and resulting local inflammatory response may enter the bloodstream, directly contributing to systemic inflammation [56]. Chronic exposure to proinflammatory cytokines, such as TNF-α and IL-1β, may alter lipid metabolism, causing hyperlipidemia [57]. TNF-α may induce insulin resistance by directly affecting target organs (e.g., liver, muscle, and adipocytes) and by indirectly promoting the production of free fatty acids from adipocytes [58]. Elevated levels of proinflammatory cytokines may also contribute to pancreatic β-cells dysfunction, leading to the development of T2DM [57,59,60,61]. Moreover, recent evidence showed that *Porphyromonas gingivalis* might induce metabolic impairment by altering the gut microbiome [62].

Our study showed inverse relationships of tooth-brushing frequency and interdental cleaning with MetS. Despite substantial heterogeneity, the findings of all subgroup analyses of tooth-brushing frequency were consistent. Tooth brushing is the most crucial self-care measure to control plaque and is a protective factor against periodontal diseases [63,64]. While a suggestion for proper frequency of tooth brushing could not be given, most of the included studies used a cut-off point of twice or more daily. Another review showed similar findings and indicated that brushing less than twice daily might not be beneficial for the prevention of DM [37]. In addition to tooth brushing, interdental cleaning is recommended for maintaining oral health. The daily use of interdental brushes was found to decrease periodontal bacteria, promote symbiotic microbiota, and reduce interdental inflammation [65]. It was suggested that poor oral hygiene could exaggerate MetS by increasing local and systemic inflammation [66].

An alternative explanation for the association between oral hygiene care and MetS might be that it is due to shared risk factors [14] or biased health consciousness. People with a healthier lifestyle might tend to adopt better oral hygiene care [67]. The fact that oral hygiene care may merely be an indicator of general health awareness or behaviors underscores the complexity of oral epidemiology [68]. However, most of the included studies in our review accounted for important confounders, such as age, gender, SES, smoking status, alcohol consumption, and physical activity, minimizing the bias.

The association between dental visits and MetS was not demonstrated in the study by Tanaka et al. [23]. This finding was similar to another study demonstrating no associations between dental visits, professional dental cleaning, and diabetes. It was argued that other confounders had more important roles in the development of diabetes than professional dental cleaning [25]. However, an earlier review has demonstrated the benefit of scaling and root planing on metabolic control and systemic inflammation reduction in patients with T2DM [69].

This systematic review and meta-analysis was the first to explore the association of oral hygiene status and care with MetS. The topic is seen as recent in the scientific literature, with the earliest identified studies published in 2009. It is also related to an emerging interest in the interrelationships between oral pathogens, oral microbiome dysbiosis, and systemic conditions [70]. Exploring this topic is relevant considering the importance of formulating policies with common risk factors approach to address both oral and general health [71]. Another strength of our review was the quality of the studies, which was moderate to high.

Our review might be limited by the methodological weakness of the included studies with a cross-sectional design. The number of cohort studies was also limited. Moreover, the restriction of studies to those published in English and the exclusion of a grey literature search might introduce bias. The risk of publication bias could not be ruled out and was not assessed in our study due to an inadequate number of studies and high heterogeneity. Besides study design and country, the potential source of heterogeneity might be from the variability in measurement methods of oral hygiene status (e.g., the use of different indices) and the reporting of tooth-brushing frequency and interdental cleaning between studies. Moreover, the criteria used to define MetS varied.

Information on tooth-brushing frequency and interdental cleaning was self-reported, which might be prone to bias. However, it might only be the type of nondifferential misclassification, leading to the underestimation of true effect estimates. Regular brushing does not necessarily reflect effective brushing, as the studies did not adjust for the duration and method of tooth brushing and the type of dentifrice used.

Finally, most of the included studies in our review were conducted among an Asian population, which may influence the generalizability of the findings worldwide. Further research conducted among other populations is warranted to provide more evidence. Using a uniform protocol for reporting oral hygiene (e.g., tooth-brushing frequency) may also facilitate better comparison.

## 5. Conclusions

Our study found that there might be inverse associations of oral hygiene status, tooth-brushing frequency, and interdental cleaning with MetS. However, substantial heterogeneity for tooth-brushing frequency and inconsistent results for oral hygiene status in subgroup analyses were observed. There was insufficient evidence on the association between dental visits and MetS. Further well-conducted studies, preferably of longitudinal design, are needed to confirm the associations of oral hygiene status and care with MetS and to explore their underlying mechanisms. Research on this topic will provide a valuable contribution to our current understanding of the interrelationship between oral health and MetS.

## Figures and Tables

**Table 2:** Subgroup analysis by study design for the association between oral hygiene status and MetS.

Subgroup	Number of Studies	OR (95% CI)	I^2^ (%)	*p*
Cross-sectional	2	0.72 (0.41–1.26)	46	0.17
Case–control	3	0.11 (0.06–0.20)	39	0.19
Cohort	1	0.83 (0.59–1.15)	-	-

MetS, metabolic syndrome; OR, odds ratio; CI, confidence interval; I^2^, percentage of variation due to heterogeneity; *p*, *p*-value for heterogeneity.

**Table 3:** Subgroup analyses for the association between tooth-brushing frequency and MetS.

Subgroup	Number of Studies	OR (95% CI)	I^2^ (%)	*p*
Study design				
Cross-sectional	5	0.67 (0.55–0.81)	93	<0.001
Cohort	2	0.74 (0.62–0.89)	0	0.64
Country				
Japan	5	0.61 (0.52–0.70)	55	0.06
Korea	2	0.85 (0.78–0.93)	73	0.06

MetS, metabolic syndrome; OR, odds ratio; CI, confidence interval; I^2^, percentage of variation due to heterogeneity; *p*, *p*-value for heterogeneity.

## Data Availability

Not applicable.

## Supplementary Materials

The following are available online at https://www.mdpi.com/article/10.3390/jcm10132873/s1, Table S1: Database search strategy, Table S2: Quality assessment of the 13 included studies.
