# Supplementary material for: Association between Oral Hygiene and Metabolic Syndrome: A Systematic Review and Meta-Analysis
Source: J Clin Med. 2021 Jun 28;10(13):2873. doi: 10.3390/jcm10132873 (PMC8269064; doi:10.3390/jcm10132873)
Supplement: Supplementary file 1 [file jcm-10-02873-s001.zip › jcm-1216397-supplementary.pdf]

Supplementary Information

Association between Oral Hygiene and Metabolic Syndrome: A Systematic Review and Meta-Analysis

Table S1. Database search strategy

**Database**		**Search strategy**	**Number of studies**
**PubMed**		("oral hygiene"[Mesh] OR "toothbrushing"[Mesh] OR "Dental Devices, Home Care"[Mesh] OR "oral hygiene" OR "tooth brush*" OR "toothbrushing" OR "interdental cleaning" OR "interdental brush*" OR floss* OR "dental visit" OR "dental attendance" OR "dental deposits"[Mesh] OR "dental plaque"[Mesh] OR "dental deposit*" OR "dental plaque" OR "oral plaque" OR "Dental plaque index"[MeSH] OR "Oral hygiene index"[MeSH] OR "plaque score" OR "oral hygiene index" OR "plaque index" OR "oral health"[Mesh] OR "mouth diseases"[Mesh] OR "oral health behavior" OR "dental health behavior" OR "oral hygiene practice") AND ("metabolic syndrome"[Mesh] OR "metabolic syndrome" OR "metabolic syndrome X" OR "Metabolic X Syndrome" OR "Cardiometabolic Syndrome" OR "Dysmetabolic Syndrome X" OR "dysmetabolic syndrome" OR "Insulin Resistance Syndrome X" OR "Metabolic Cardiovascular Syndrome" OR "Reaven Syndrome X" OR "Syndrome X" OR "insulin resistance syndrome" OR "plurimetabolic syndrome") Filters: English	271
**Web of Science**	1	ALL FIELDS: (“oral hygiene” OR “tooth brush*” OR “toothbrushing” OR “interdental cleaning” OR “interdental brush*” OR floss* OR “dental visit” OR “dental attendance” OR “dental deposit*” OR “dental plaque” OR “oral plaque” OR “plaque score” OR “oral hygiene index” OR “plaque index” OR “oral health behavior” OR “dental health behavior” OR “oral hygiene practice”) OR TOPIC: (“oral health” OR “mouth disease*”)<br>Indexes=SCI-EXPANDED, SSCI, A&HCI, CPCI-S, CPCI-SSH, BKCI-S, BKCI-SSH, ESCI, CCR-EXPANDED, IC Timespan=All years	59,826
	2	ALL FIELDS: (“metabolic syndrome” OR “metabolic syndrome X” OR “Metabolic X Syndrome” OR “Cardiometabolic Syndrome” OR “Dysmetabolic Syndrome X” OR "dysmetabolic syndrome" OR “Insulin Resistance Syndrome X” OR “Metabolic Cardiovascular Syndrome” OR “Reaven Syndrome X” OR “Syndrome X” OR “insulin resistance syndrome” OR “plurimetabolic syndrome”)<br>Indexes=SCI-EXPANDED, SSCI, A&HCI, CPCI-S, CPCI-SSH, BKCI-S, BKCI-SSH, ESCI, CCR-EXPANDED, IC Timespan=All years	109,997
	3	#1 AND #2<br>Refined by: LANGUAGES: (ENGLISH)<br>Indexes=SCI-EXPANDED, SSCI, A&HCI, CPCI-S, CPCI-SSH, BKCI-S, BKCI-SSH, ESCI, CCR-EXPANDED, IC Timespan=All years	233

Note: The date of the last search was March 17, 2021.

Table S2. Quality assessment of the 13 included studies.

Study	Study design	Selection	Comparability	Outcome / Exposure	Total score
Fukui et al, 2012 [1]	Cross-sectional	1	2	2	5 / 8
Kim et al, 2013 [2]	Cross-sectional	2	2	2	6 / 8
Tsutsumi and Kakuma, 2015 [3]	Cross-sectional	0	2	2	4 / 8
Kim et al, 2019 [4]	Cross-sectional	2	2	2	6 / 8
Saito et al, 2019 [5]	Cross-sectional	1	2	2	5 / 8
Shearer et al, 2018 [6]	Cross-sectional	3	2	2	7 / 8
Chen et al, 2011 [7]	Cross-sectional	1	2	2	5 / 8
Kobayashi et al, 2012 [8]	Cohort	2	2	3	7 / 9
Tanaka et al, 2018 [9]	Cohort	2	2	3	7 / 9
Pussinen et al, 2020 [10]	Cohort	3	2	3	8 / 9
Pham, 2018 [11]	Case-control	3	1	1	5 / 9
Li et al, 2009 [12]	Case-control	3	2	2	7 / 9
Li et al, 2020 [13]	Case-control	3	2	2	7 / 9

Note: The quality of the included studies was assessed using Newcastle-Ottawa Scale for cross-sectional, case-control, and cohort studies, as applicable. The maximum score for cross-sectional studies was 8 points (4 for selection, 2 for comparability, and 2 for outcome). The maximum score for case-control studies was 9 points (4 for selection, 2 for comparability, and 3 for exposure). The maximum score for cohort studies was 9 points (4 for selection, 2 for comparability, and 3 for outcome) [14,15].

References

1. Fukui, N.; Shimazaki, Y.; Shinagawa, T.; Yamashita, Y. Periodontal Status and Metabolic Syndrome in Middle-Aged Japanese. *J. Periodontol.* **2012**, *83*, 1363–1371, doi:10.1902/jop.2012.110605.

2. Kim, Y.H.; Kim, D.H.; Lim, K.S.; Ko, B.J.; Han, B.D.; Nam, G.E.; Park, Y.G.; Han, K.D.; Kim, J.H.; Cho, K.H. Oral health behaviors and metabolic syndrome: The 2008-2010 Korean National Health and Nutrition Examination Survey. *Clin. Oral Investig.* **2014**, *18*, 1517–1524, doi:10.1007/s00784-013-1112-2.

3. Tsutsumi, C.; Kakuma, T. Regular Tooth Brushing is Associated with a Decreased Risk of Metabolic Syndrome According to a Medical Check-Up Database. *Kurume Med. J.* **2015**, *61*, 43–52, doi:10.2739/kurumemedj.MS64004.

4. Kim, J.S.; Kim, S.Y.; Byon, M.J.; Lee, J.H.; Jeong, S.H.; Kim, J.B. Association between periodontitis and metabolic syndrome in a korean nationally representative sample of adults aged 35–79 years. *Int. J. Environ. Res. Public Health* **2019**, *16*, 2930, doi:10.3390/ijerph16162930.

5. Saito, M.; Shimazaki, Y.; Nonoyama, T.; Tadokoro, Y. Number of teeth, oral self-care, eating speed, and metabolic syndrome in an aged Japanese population. *J. Epidemiol.* **2019**, *29*, 26–32, doi:10.2188/jea.JE20170210.

6. Shearer, D.M.; Thomson, W.M.; Cameron, C.M.; Ramrakha, S.; Wilson, G.; Wong, T.Y.; Williams, M.J.A.; McLean, R.; Theodore, R.; Poulton, R. Periodontitis and multiple markers of cardiometabolic risk in the fourth decade: A cohort study. *Community Dent. Oral Epidemiol.* **2018**, *46*, 615–623, doi:10.1111/cdoe.12414.

7. Chen, L.-P.; Hsu, S.-P.; Peng, Y.-S.; Chiang, C.-K.; Hung, K.-Y. Periodontal disease is associated with metabolic syndrome in hemodialysis patients. *Nephrol. Dial. Transplant.* **2011**, *26*, 4068-4073, doi:10.1093/ndt/gfr209.

8. Kobayashi, Y.; Niu, K.; Guan, L.; Momma, H.; Guo, H.; Cui, Y.; Nagatomi, R. Oral health behavior and metabolic syndrome and its components in adults. *J. Dent. Res.* **2012**, *91*, 479–484, doi:10.1177/0022034512440707.

9. Tanaka, A.; Takeuchi, K.; Furuta, M.; Takeshita, T.; Suma, S.; Shinagawa, T.; Shimazaki, Y.; Yamashita, Y. Relationship of toothbrushing to metabolic syndrome in middle-aged adults. *J. Clin. Periodontol.* **2018**, *45*, 538–547, doi:10.1111/jcpe.12876.

10. Pussinen, P.J.; Paju, S.; Viikari, J.; Salminen, A.; Taittonen, L.; Laitinen, T.; Burgner, D.; Kahonen, M.; Lehtimaki, T.; Hutri-Kahonen, N.; et al. Childhood Oral Infections Associate with Adulthood Metabolic Syndrome: A Longitudinal Cohort Study. *J. Dent. Res.* **2020**, *99*, 1165–1173, doi:10.1177/0022034520929271.

11. Pham, T. The association between periodontal disease severity and metabolic syndrome in Vietnamese patients. *Int. J. Dent. Hyg.* **2018**, *16*, 484–491, doi:10.1111/idh.12350.

12. Li, P.; He, L.; Sha, Y.Q.; Luan, Q.X. Relationship of Metabolic Syndrome to Chronic Periodontitis. *J. Periodontol.* **2009**, *80*, 541–549, doi:10.1902/jop.2009.080387.

13. Li, P.; He, L.; Chen, Z.B.; Luan, Q.X. Biomarkers in Metabolic Syndrome Patients with Chronic Periodontitis. *Chin. J. Dent. Res.* **2020**, *23*, 191–197, doi:10.3290/j.cjdr.a45223.

14. Wells, G.; Shea, B.; O'Connell, D.; Peterson, J.; Welch, V.; Losos, M.; Tugwell, P. The Newcastle-Ottawa Scale (NOS) for Assessing the Quality of Nonrandomised Studies in Meta-Analyses. Available online: http://www.ohri.ca/programs/clinical_epidemiology/oxford.asp (accessed on 5 February 2021).

15. Yuan, T.; Zou, H.; Zhao, J.; Yang, Z.; Li, L.; Cai, W.; Gu, J.; Hao, C.; Li, J.; Hao, Y.; et al. Circumcision to prevent HIV and other sexually transmitted infections in men who have sex with men: A systematic review and meta-analysis of global data. *Artic. Lancet Glob. Health* **2019**, *7*, e436–e447, doi:10.1016/S2214-109X(18)30567-9.
